# *The Organon* and Materia Medica Club of the Bay Cities of California

**Published:** 1896-10

**Authors:** W. E. Ledyard

**Affiliations:** Secretary


### *THE ORGANON* AND MATERIA MEDICA CLUB OF THE BAY CITIES OF CALIFORNIA.

The regular semi-monthly meeting was held at the office of Dr. George H. Martin, 606 Sutter Street, San Francisco, Friday evening, May 1st, 1896.

Members present: Drs. J. M. Selfridge, W. E. Ledyard, A. McNeil, George H. Martin, and M. T. Wilson.

The meeting was called to order at 8.30 o'clock by the President, Dr. J. M. Selfridge. The minutes of the previous meeting were read by the Secretary and, after corrections by Drs. Selfridge and Ledyard, were approved.

Dr. Wilson presented a case of a boy three years of age in whom there was a peculiar condition of the sternum and ribs. Dr. Wilson considered it a case of imperfect ossification, and wished the opinions of the other physicians present. After examination of the boy all seemed to agree that it was a case of rickets.

The President appointed Dr. McNeil reader of the evening, who commenced *The Organon* at Section 130, 131.

### Discussion.

Dr. Selfridge—This certainly proves what Hahnemann claims, that a drug will produce symptoms in a well person that can be cured in a sick person by the same drug.

Dr. McNeil—There is one point on the question of proving, which is that we do not always know beforehand whether the person will be sensitive to the effect of the drug or not. If the person is not sensitive to the drug we would have to repeat the dose.

Dr. Ledyard—This may throw some light upon the question of one dose proving fatal in some cases. One dose of *Phosphorus* in tuberculosis may cause an aggravation, and then another dose is given to overcome this aggravation. I have noticed this aggravation, but do not repeat the dose.

Dr. McNeil—H. C. Allen and others say that *Sulphur*, *Phosphorus*, *Arsenicum*, and *Silicea* should not be given, even when indicated, in the last stages of consumption. I have seen several cases diagnosed as tuberculosis by other physicians, and I have given these antipsorics and had benefit. One case I remember I kept the patient in fair health for ten years, and one of the Presidents of the American Institute of Homœopathy said that he could not live four months.

Dr. Selfridge—I cannot agree with Allen and others, though I do not like to question the opinions of such men.

Dr. McNeil—They may be mistaken with regard to the condition of the patient. The disease is liable to terminate suddenly. What evidence have we that after giving the indicated remedy the patient died from the effect of that remedy?

Dr. Martin—There is apt to be too much stress laid upon the effect of the remedy, either for good or evil. You have a fatal disease, liable to end at any time, and it is merely a coincidence if the end comes after giving the remedy.

Dr. Ledyard—Have you not seen a dangerous aggravation from *Phosphorus?*

Dr. McNeil—No. It may only be a coincidence. This happens usually with men who use the very high potencies. If this is so, it tells us that we should avoid the highest potencies rather than the drug.

Dr. Ledyard—I had a case in which I gave *Phosphorus*, and the patient died shortly afterward. Death followed so closely after giving the remedy that I thought it was the cause.

Dr. McNeil—Had you given the patient *Phosphorus* before?

Dr. Ledyard—Yes, and *it did good.* When I gave it toward the last it was plain to be seen that the patient was very much worse. The vital force was not sufficiently strong to bring about a healthy reaction, and in the effort to restore the equilibrium, the patient succumbed.

Dr. Selfridge—I had one case of consumption in which the patient had become cold and cyanosed. I was sure she would not live the night out. She and her husband commenced to talk over the children, etc., and the wife became *angry* and lived four days.

Dr. Martin—It is a mistake to make a prognosis as to time.

Sections 132, 133 were read.

### Discussion.

Dr. McNeil—This last paragraph is one that excites the non-Hahnemannian to a high degree. He says it is nonsense. We all know that it is not nonsense. I recall one case that died, and I know if the patient had received *Phosphorus* at the right time she would have lived.

Dr. Selfridge—I have often noticed that the modalities are often the keynotes of the remedy.

Dr. McNeil—If you strip a case of its modalities you rob it of its originality.

Sections 134–136 were read.

### Discussion.

Dr. McNeil—Painless morning diarrhœa of *Sulphur* occurred only in one prover, Franz Hahnemann. Hempel left out this symptom, and it is a valuable one. Allen's *Encyclopædia* shows that some of our most valuable symptoms occurred but once.

Dr. Ledyard—That is *very peculiar* for a symptom to occur only in one person, and *it is the peculiar symptoms which are characteristic.*

Dr. McNeil—The individuality then comes out.

Dr. Selfridge—What an acute observer Hahnemann was to notice all these things. He was a wonderful man.

Dr. Martin—He was truly a wonderful man to think these things out himself for the first time.

Section 137 was read.

DISCUSSION.

Dr. McNeil—Here is a point : *the necessity of going to the higher potencies in order to get the finer shades of symptoms.* I do not believe that a chemist or toxicologist could tell whether a case of poisoning was due to Belladonna or Stramonium, the symptoms are so much alike. Provings by potencies bring out the difference.

Dr. Ledyard—Both have desire for and dread of light. *Stramonium* has a much greater desire for light than *Belladonna*, while *Belladonna*, on the other hand, has a much greater dread of light than *Stramonium.*

Sections 138–141 were read.

The meeting was then declared adjourned, to meet again the first Friday in June instead of the third Friday in May, as the State Society meeting would take place at that time. At the next meeting the reading of *The Organon* would be commenced at Section 142.

W. E. LEDYARD, *Secretary.*

Reported by Eleanor F. Martin, M. D.

